# Finding Meaning in Hell. The Role of Meaning, Religiosity and Spirituality in Posttraumatic Growth During the Coronavirus Crisis in Spain

**DOI:** 10.3389/fpsyg.2020.567836

**Published:** 2020-11-05

**Authors:** María Prieto-Ursúa, Rafael Jódar

**Affiliations:** Department of Psychology, Faculty of Social and Human Sciences, Universidad Pontificia Comillas, Madrid, Spain

**Keywords:** posttraumatic growth, meaning in life, spirituality, religiosity, coronavirus, COVID-19

## Abstract

Coronavirus has blighted our world, hitting some countries harder than others. Morbidity and mortality rates make Madrid one of the worst affected places so far in the wake of the coronavirus. The aim of this study was to analyze the presence of post-traumatic growth during the coronavirus crisis and to understand the contribution of meaning, religiosity, and spirituality to such growth; 1,492 people completed the questionnaire; *N* = 1,091 residents in Madrid were selected for the study. We assessed the personal experience of COVID-19, the Spirituality, Religiosity, Meaning trough Purpose in Life-10 test, and Posttraumatic Growth (Community Post-Traumatic Growth Scale). Results showed significant differences for all measures of growth, with higher values in women. Sex and direct impact of COVID-19 accounted for 4.4% of the variance of growth. The different dimensions of meaning contribute differently to growth. Only religiosity was associated with total growth when meaning was included in the model. This same pattern of results is obtained in models predicting interpersonal and social growth. However, in predicting personal growth, it is spirituality that predicts this type of growth once meaning has been previously controlled for, while religiosity fails to reach a statistically significant level. Our results reflect the interest in maintaining the distinction between spirituality and religiosity, their different roles in traumatic growth and the different dimensions on which each has an effect. Finally, it confirms the importance of meaning in post-traumatic growth, especially the dimension of life goals and purposes.

## Introduction

Coronavirus has blighted our world, hitting some countries harder than others. At the time of writing (May 2020), Spain had 282,852 cases (6,050 cases per million inhabitants) and more than 28,750 deaths. Twenty-five percent of cases (67,932) and 31% of deaths (8,977) nationwide are concentrated in the capital, Madrid, with a population of 6,662,000 inhabitants (14% of national population). When lockdown was decreed on March 14, 180 people had been admitted to ICUs in Madrid for COVID-19; three weeks later, ICU admissions stood at 1,528, tripling the city’s healthcare capacity. Morbidity and mortality rates make Madrid (along with Lombardy, Paris, or New York) one of the worst affected places so far in the wake of the coronavirus, following statistical data^1^. The lockdown in Spain was one of the strictest and compliance levels were very high^2^.

The overloaded healthcare system also affected family experiences of hospitalization and the death of loved ones. The sick were alone in hospital with no loved ones to accompany them and died with nobody they knew by their side. It is true that our healthcare personnel showed great professionalism and compassion in bringing tenderness and humanity at this time, but the distance, loneliness and lack of contact, burials with only two people per deceased allowed, and the lockdown of relatives made it much harder to mourn and access social support.

Different algorithms^3^ have shown that on the one hand, the astonishing number of patients and deaths in Madrid can be explained by the delay in decreeing a state of emergency. However, while its preventive effect was minimal, it did serve to decrease the enormous pressure on healthcare services, which was very important in preventing an even greater number of deaths.

Thus, alongside the situation of threat, suffering, illness, and death that Madrid has gone through, and the tough conditions experienced by healthcare personnel and citizens alike, we have to add the difficulty of finding answers and the general perception among the population of “being late,” of the uselessness of the measures taken, and of the idea that, although part of the suffering was inevitable, another part could have been avoided with earlier action.

However, even in these circumstances, psychology suggests that positive aspects can be found, for example personal change and growth, whether in social or spiritual terms. In 1999, Calhoun and Tedeschi defined post-traumatic growth as the subjective experience of positive psychological change reported by a person as a result of struggling with a traumatic event. As Páez et al. (2012) point out, traumatic events can have positive effects on a personal level (increasing wisdom and knowledge about oneself and others, appreciation of what one has and learning important priorities in life), in relationships with others (bringing the family together and keeping them closer, uniting the community, being more tolerant and compassionate with others, valuing the support they offer). Social growth can also be observed (reinforcing positive beliefs about the group, perceiving growth in the community), as can spiritual growth (having a better understanding of spiritual matters, having a stronger religious faith - Calhoun and Tedeschi, 1999).

Several factors contribute to facilitate growth. One of them is making sense of experience and existence itself. It is precisely by confronting this type of situation, showing the transitory nature of our existence and the inevitability of suffering (the “primordial facts,” according to Frankl), that the opportunity for discovering meaning in life presents itself (Linley and Joseph, 2011). Triplett et al. (2012) suggest that repeated thinking or “rumination” leads to the assimilation of the situation to existing cognitive structures and the development of fundamental beliefs which make it possible to accommodate experience successfully. The presence of meaning is central to understanding positive change, which emerges after adversity through cognitive and emotional processing that reconstructs one’s vision of self and the world (Schultz et al., 2010; Linley and Joseph, 2011).

Meaning can encompass different dimensions. García-Alandete et al. (2013) propose that these include the meaning given to experience or existence, that is, the understanding of experience within a broader context and personal satisfaction with life. Furthermore, meaning is also expressed in having a direction, aims, a vital purpose, a mission toward the achievement of which the individual directs his or her efforts. According to these authors, the first dimension has a more cognitive character, while the second is more motivational.

Other factors that facilitate growth are spirituality and religiosity. Many studies have found clear relationships between these variables and post-traumatic growth (see, for example, the meta-analysis by Shaw et al., 2005). However, some conceptual issues should be clarified regarding these variables in the context of post-traumatic growth. First of all, spiritual growth and the deepening of religious beliefs are conceived as a dimension of growth, as a result of growth; thus, studying their presence or their correlation with growth experiences can lead to a conceptual “overlap”, since they are part of the concept to which they are linked (McGrath, 2011). It has therefore been recommended that spirituality or religiosity should not be included in measures of post-traumatic growth (Joseph, 2011). In addition, Shaw et al. (2005) point out that most studies have been correlational, and that it would be necessary to study in depth the extent to which these variables are antecedent or consequent. Third, spirituality and religiosity are not usually differentiated, but rather included in a single concept “spirituality/religiosity”, or both terms are used interchangeably. Although related, they are not synonymous. According to Pargament (1997), spirituality refers to the fundamental function of religion as *the search for the sacred and the meaning of life*, and religiosity may be defined as the degree of commitment to the characteristic beliefs and practices of a particular religious tradition. For Hill et al. (2000), both constitute experiences of searching for the sacred. The differentiating characteristics are that religiosity: 1) can also include the search for non-sacred values, such as security, personal well-being, affiliation – values found in contexts normally focused on searching for the sacred; 2) legitimizes the search for the sacred by a group through their structures (practices, rituals). Spirituality, meanwhile, would be a more privatized and less normative experience.

Many people define their personal spirituality as something different from any religion, so four options could be established in relation to religion and spirituality (Zinnbauer et al., 1997): being both religious and spiritual, being religious but not spiritual, being spiritual but not religious or being neither religious nor spiritual. As Shaw et al. (2005) argue, it is important to direct efforts toward a clarification of the differences in their respective contribution to growth. Since the cognitive processes involved in growth are centered on great existential questions about meaning, questions which are also central to spirituality (McGrath, 2011), a greater contribution of spirituality to growth would be expected (Shaw et al., 2005).

The aim of this study was to analyze the presence of post-traumatic growth during the coronavirus crisis and to understand the contribution of meaning, religiosity and spirituality to such growth. Specifically, we expect to find more growth linked to the dimension of meaning and sense in life (rather than the dimension of aims and purposes in life) and to greater spirituality.

## Materials and Methods

### Participants and Procedure

A total of *N* = 1,091 residents in Madrid completed the questionnaire, 69.4% of whom were women (757). The sample was divided into age groups, with 34.4% aged 19–29 years, 8.1% aged 30–39 years, 22.0% aged 50–59 years, 18.1 aged 40–49 years, and 16.5% aged over 60 years. Sixty-seven percent of the sample were university graduates and 79.8% reported that their pre-crisis economic situation was good or very good.

Surveys were designed using Google Forms and distributed online, through LinkedIn, e-mail, and WhatsApp, to as wide a range of participants as possible through the researchers’ social and work networks using a snowball sampling technique. The questionnaire was available in Spanish, and participants were anonymous, although they were offered the possibility of providing their email if they wished to be informed of the general results of the study. The only inclusion criteria were being of legal age (i.e., over 18 years old) and resident in Spain during lockdown. Data were collected during May 2020, the final month of lockdown.

### Instruments

*Sociodemographic Data.* We assessed age range, according to age groupings used by the Ministry of Health, sex, highest educational level attained and perception of the economic situation before the crisis (with five categories: very bad, bad, fair, good or very good).

*Perceived Spirituality and Perceived Religiosity*. Perceived Spirituality was measured with the item *To what extent do you consider yourself a spiritual person*?, and Perceived Religiosity with the item *To what extent do you consider yourself a religious person?*. Following Krause et al. (2020), we decided against offering a definition of each term. Both questions had a 5-item response scale ranging from 1: not at all to 5: profoundly or extremely. Scores of 4 and 5 signaled High Perceived Religiosity (R+) and High Perceived Spirituality (S+), while scores of 1 and 2 signaled Low Perceived Religiosity (R−) and Low Perceived Spirituality (S−).

*Experience of COVID-19.* To ascertain the subject’s direct contact with the disease, the following questions were asked: *Have you been diagnosed with coronavirus?* (Respondents were asked to differentiate between diagnosis by test, by a doctor or by self-diagnosis based on own symptoms). *Have you been hospitalized for coronavirus? Have you been admitted to the ICU for coronavirus? Have any of your loved ones been hospitalized for coronavirus? Have any of your loved ones been admitted to the ICU for coronavirus? Did you personally know anyone who has died of coronavirus? Have any of your loved ones died of coronavirus? Do you know anyone with a family member who died of coronavirus?* All items had a dichotomous response option, YES or NO.

*Purpose In Life Test-10 (PIL-10*, García-Alandete et al., 2013). *The Purpose In Life Test (PIL*, Crumbaugh and Maholick, 1969) is one of the most widely used instruments in meaning research, in particular part A, a scale of 20 Likert-type items, with 7 response categories. Although the authors of the instrument proposed it as a one-dimensional scale, different factors have been found in different studies. The present study follows the proposal of García-Alandete et al. (2013), who studied its psychometric properties in the Spanish population and proposed a reduction to a 10-item scale with a structure of two correlated factors: a cognitive-evaluative factor, related to the perception and general assessment of the meaning of life (Satisfaction and Sense of Life, SSL) and a motivational factor, related to the establishment of specific goals and vital purposes (Goals and Purposes in Life, GPL). Scores on the SSL scale range from 1 to 43, those on the GPL scale from 1 to 28, with a total score between 2 and 70, where higher scores indicate a higher level of meaning. In our sample, this questionnaire achieved internal consistency (Cronbach’s alpha) of 0.899, with the GPL and SSL subscales showing Cronbach’s alpha of 0.799 and 0.858, respectively.

*Community Post-Traumatic Growth (CPTG)* (Páez et al., 2012). The scale has 24 items with a range of responses from 1 (strongly agree) to 5 (strongly disagree). It investigates post-traumatic growth at four levels: personal (example item: *I have changed my priorities about what is important in life*), interpersonal (e.g., *I have discovered the support of people who were not close to me*), social (e.g., *I have discovered that my community, group, family is stronger than I thought*) and socio-political participation (e.g., *Political and ethical participation and commitments in the country have increased*). Our study assessed only the first three levels. Each subscale has a scoring range of 1 to 30 points, with a total scoring range, in our study, of between 3 and 90 points, a greater score pointing to higher levels of growth. In our sample, this questionnaire achieved internal consistency (Cronbach’s alpha) of 0.928 (Total Growth), and Cronbach’s alpha for the subscales of 0.861 for Personal Growth, 0.871 for Interpersonal Growth, and 0.830 for Social Growth.

### Data Analysis

Given that the distribution of the variables lacked normality, confidence interval estimates were made using bootstraps, with 10,000 samples. Non-parametric tests were used to estimate differences between groups in terms of growth, meaning, Perceived Spirituality, and Perceived Religiosity, and the Bonferroni correction was used for *post hoc* planned comparisons. Likewise, bootstrapping was also used in the hierarchical regression models to estimate the confidence intervals. Hierarchical regression analysis was used to explore the amount of variance of post-traumatic growth exclusively associated with perceived spirituality and religiosity. The statistical procedures were computed using the SPSS Package (v.24; SPSS Inc., Chicago, IL, United States).

## Results

One hundred and nine (10%) of the participants had been diagnosed with coronavirus, 6 of them had been hospitalized for coronavirus (one of them in the ICU), 254 participants (23.3%) had loved ones hospitalized for coronavirus (120 in ICU), more than half of the sample knew someone who had died of coronavirus personally (590 people, 54.1%), 143 people had lost a loved one to coronavirus (13.1%) and 80.1% (874 people) knew someone who had lost a family member to coronavirus.

Table 1 shows the mean scores for meaning, growth, Perceived Spirituality and Perceived Religiosity of the sample.

**TABLE 1:** Average scores in Sense, Growth, Perceived Spirituality, and Perceived Religiosity.

**Variables**	***N***	**Min MAX**	**Mean**	**SD**	**LL 95% CI**	**UL 95% CI**
Total growth	1075	1-5	3.18	0.81	3.13	3.23
Personal	1075	1-5	3.13	0.94	3.08	3.18
Interpersonal	1075	1-5	3.3	0.91	3.24	3.35
Social	1075	1-5	3.11	0.89	3.06	3.16
Total meaning	1075	1.7-7	5.29	0.94	5.23	5.35
SSL	1075	1.17-7	5.04	1.05	4.97	5.1
GPL	1075	2-7	5.67	0.95	5.61	5.73
Perceived Spirituality	1075	1-5	3.32	1.09	3.25	3.38
Perceived Religiosity	1075	1-5	2.75	1.35	2.67	2.83

*16 participants had missing data. SSL, Satisfaction and Sense of Life; GPL, Goals and Purposes in Life; LL UL, Lower and Upper Limit, 95% confidence interval, bootstrap estimation.*

Table 2 presents the values for men and women in meaning and growth. Differences between men and women were not found in SSL (Mann-Whitney *U* = 122956.5, *p* = 0.611) but did exist in GPL, being higher in women (*U* = 108272, *p* = 0.002). There are no differences between men and women in total meaning (*U* = 118013.5, *p* = 0.337).

**TABLE 2:** Values for meaning and growth in men and women.

		**Women**					**Men**				
**Variables**				**Bootstrap 95% CI**					**Bootstrap 95% CI**		**Mann-Whitney U**
	**Average rank**	**Mean**	**SD**	**LL**	**UL**	**Average rank**	**Mean**	**SD**	**LL**	**UL**	***p***
Total meaning	544.0	5.3	0.96	5.23	5.37	524.3	5.26	0.92	5.16	5.36	0.337
SSL	549.3	5.02	1.01	4.94	5.1	550.8	5.08	1.01	4.97	5.19	0.611
GPL	558.4	5.72	0.95	5.65	5.79	494.6	5.54	0.93	5.44	5.64	0.002
Total growth	573.6	3.25	0.79	3.19	3.3	483.5	3.03	0.82	2.94	3.12	< 0.001
Personal	584.8	3.24	0.93	3.17	3.3	458.0	2.89	0.90	2.79	2.99	< 0.001
Interpersonal	565.2	3.35	0.92	3.29	3.42	502.5	3.18	0.90	3.08	3.28	0.002
Social	559.4	3.15	0.88	3.09	3.22	515.7	3.02	0.93	2.92	3.12	0.034
Spirituality	576.8	3.42	1.07	3.35	3.5	476.3	3.07	1.11	2.96	3.19	< 0.001
Religiosity	554.4	2.78	1.35	2.69	2.88	527.0	2.67	1.35	2.53	2.82	0.174

*Statistically significant differences were found for all measures of growth (total, personal, interpersonal, and social) (*U* = 105534.0, *p* < 0.001 for total; *U* = 97034.5, *p* < 0.001 for personal; *U* = 111896.5, *p* = 0.002 for interpersonal; and *U* = 116286.5, *p* = 0.035 for social) always with higher values in women. SSL, Satisfaction and Sense of Life; GPL, Goals and Purposes in Life.*

Regarding the relationship between Perceived Spirituality and Perceived Religiosity, while there are no differences in Perceived Religiosity (*U* = 120081.5, *p* = 0.174), women have higher scores in Perceived Spirituality (*U* = 103124.5, *p* < 0.001).

We then grouped the subjects into the four possible categories according to their Perceived Religiosity (R+ or R-) and Perceived Spirituality (S+ or S-) scores. Subjects with intermediate scores (3) were not included in the analyses. Subjects were grouped as follows: 346 subjects (31.7%) in the R+ S+ group, 8 (0.7%) in the R+ S− group, 116 (10.6%) in the R− S+ group, and 216 (19.8%) in the R–S group.

Since there were not enough people in the R+ S− group, the other three were compared (Table 3). We found differences between these groups in all growth variables (Kruskal–Wallis H for total, personal, interpersonal and social growth was *H* = 74.43, *H* = 53.53, *H* = 49.31, and *H* = 68.97, respectively, with *p* < 0.001 in all four cases). Planned comparisons with Bonferroni correction revealed greater total, personal, interpersonal and social growth in the R+ S+ group than in the R− S− group (*U* = 21389.5, *U* = 23920.5, *U* = 24401.0, and *U* = 22518.5, respectively; *p* < 0.001 in all cases). The R+ S+ group showed higher values than the R− S+ group in total (*U* = 16516.5, *p* = 0.004) and social growth (*U* = 14324.5, *p* < 0.001), but not in personal (*U* = 19037.0, *p* > 0.013) or interpersonal growth (*U* = 17083.5, *p* > 0.013). Finally, in measures of growth, the R− S+ group exceeded the R-S- group in total (*U* = 9057.5, *p* < 0.001), personal (U = 8741.0, *p* < 0.001) and interpersonal growth (*U* = 9747.0, *p* < 0.001), but not in social growth (*U* = 10800, *p* > 0.013).

**TABLE 3:** Comparison between the three religious groups.

	**R− S−**					**R− S+**					**R+ S+**				
**Variables**				**Bootstrap 95% CI**					**Bootstrap 95% CI**					**Bootstrap 95% CI**	
	**Average rank**	**Mean**	**SD**	**LL**	**UL**	**Average rank**	**Mean**	**SD**	**LL**	**UL**	**Average rank**	**Mean**	**SD**	**LL**	**UL**
Total meaning	247.0	4.87	1.06	4.72	5.01	339.1	5.35	0.98	5.16	5.52	387.3	5.61	0.80	5.53	5.69
SSL	254.2	4.60	1.15	4.45	4.74	349.4	5.12	1.08	4.92	5.31	386.8	5.36	0.97	5.26	5.45
GPL	257.1	5.28	1.1	5.13	5.43	328.7	5.70	0.98	5.52	5.87	385.6	5.99	0.79	5.91	6.07
Total growth	249.5	2.83	0.80	2.72	2.93	338.8	3.15	0.78	3.01	3.29	396.0	3.40	0.78	3.32	3.48
Personal	259.7	2.73	0.89	2.61	2.85	363.3	3.18	0.99	3.01	3.36	381.4	2.73	0.89	2.61	2.85
Interpersonal	266.6	2.98	0.94	2.86	3.11	337.8	3.32	0.86	3.17	3.48	385.6	3.52	0.88	3.43	3.60
Social	262.8	2.77	0.93	2.65	2.90	304.9	2.95	0.89	2.79	3.14	399.0	3.38	0.86	3.29	3.47

*SSL, Satisfaction and Sense of Life; GPL, Goals and Purposes in Life.*

Differences were also found in all the meaning variables (Table 3), with Kruskal–Wallis H for total meaning, SSL and GPL of *H* = 69.26, *H* = 61.97, and *H* = 58.46, respectively, and *p* < 0.001 in all three cases. Planned-comparisons with Bonferroni correction yielded greater total meaning, SSL and GPL in the R+ S+ group than in the R− S− group (*U* = 21089.5, *U* = 22623.5, and *U* = = 22296.0 for total meaning, SSL and GPL; *p* < 0.001 in all three cases). The R+ S+ group showed higher values than the R− S+ group in total meaning (*U* = 16653.5, *p* < 0.017), in GPL (*U* = 16371.0, *p* = 0.005), but not in SSL (*U* = 17524.0, *p* = 0.066). Finally, the R− S+ group exceeded the R− S− group in all meaning measurements: total (*U* = 8702.0, *p* < 0.001), SSL (*U* = 8851.5, *p* < 0.001), and GPL (*U* = 9624.0, *p* < 0.001).

We computed hierarchical regression models to explore the prediction of growth from meaning, religiosity and spirituality, once sociodemographic and the impact of COVID is taken into account. Table 4 shows the results with total growth as the dependent variable (analyses on subscales can be consulted as Supplementary Tables 1–3). Sex and age were initially entered as independent variables. In a second model, dichotomous variables which included the direct impact of COVID-19 were added. These two sets of variables accounted for 4.4% of the variance of growth. Once the effect of sex, age and the impact of COVID-19 were taken into account, the meaning variables were introduced; as we can see, only GPL, not SSL, manages to account for growth. In a final step, the variables of Perceived Religiosity and Perceived Spirituality were introduced. Only Perceived Religiosity predicts total growth in a statistically significant way if meaning has been previously taken into account. This same pattern of results is obtained in models predicting interpersonal and social growth. However, in predicting personal growth, it is Perceived Spirituality that predicts this type of growth once meaning has been previously controlled for (*B* = 0.45, SE = 0.22, *p* = 0.044, 95% CI = [0.016 0.89]), while Perceived Religiosity fails to reach a statistically significant level (*B* = 0.34, SE = 0.17, *p* = 0.050, 95% CI = [−0.01 0.67]).

**TABLE 4:** Hierarchical regression models of total growth, on meaning and Perceived Religiosity and Perceived Spirituality (controlled by age, sex and impact of COVID-19).

		**Total growth**		**95% CI**	
**Predictor**	**Δ*R*^2^**	**β**	**SE**	**Lower limit**	**Upper limit**
Step 1	0.022***				
Age		0.79**	2.22	0.20	1.36
Sex		4.68**	1.07	2.65	6.78
Step 2	0.022***				
Diagnosed with coronavirus		1.81**	0.65	0.53	3.06
With loved ones in hospital		1.13	1.43	−1.74	3.88
With loved ones in ICU		−2.24	1.99	−6.06	1.71
Knowing people who died		3.85***	1.01	1.85	5.77
With loved ones who died		0.075	1.65	−3.23	3.28
Step 3	0.077***				
SSL		−0.14	0.71	−1.51	1.26
GPL		4.61***	0.71	3.17	6.03
Step 4	0.026***				
Perceived Spirituality		0.92	0.58	−0.21	2.1
Perceived Religiosity		1.38**	0.43	0.52	2.24
Total *R*^2^	0.147***				

SSL, Satisfaction and Sense of Life; GPL, Goals and Purposes in Life. *p < 0.05; **p < 0.01; ***p < 0.001.

## Discussion

First, we can confirm that even in traumatic and life-threatening situations, such as that caused by the coronavirus in Madrid, it is possible to find indicators of positive growth at various levels of experience, which bears witness to the resilience of human beings and their enormous capacity to overcome problems. These results are congruent with those found by López et al. (2020) regarding well-being (growth and meaning) in old people in Spain during lockdown: even the most threatened age group managed to find personal resources for growth.

In all growth dimensions, higher values were found among women, results that coincide with the available evidence (see, for example, the meta-analysis by Vishnesky et al., 2010). Apart from differences, our study also shows that being a woman contributes significantly to post-traumatic growth, a finding which encourages us to continue exploring the underlying processes that explain this function; a possible hypothesis suggests that women’s greater tendency to ruminative thinking or their style of coping, centered on emotion, facilitates the growth process (Vishnesky et al., 2010).

Direct contact with disease and death in this crisis is shown to be associated with the variables studied. Having known someone who died predicts greater personal, interpersonal and social growth, and greater Perceived Religiosity. Having been diagnosed with COVID-19 also predicts further growth, at all levels. It seems, then, that close contact with death or with the personal experience of the possibility of death, mobilizes resources which are necessary to focus attention on the positive aspects of the experience.

It is also interesting to see that the different dimensions of meaning contribute differently to growth. Having vital goals and purposes, being willing to reach goals and achievements, are associated with post-traumatic growth to a greater extent than experiencing that life is meaningful, valuable, or exciting.

Perhaps the most interesting results of our study are those showing the different contributions of Perceived Religiosity and Perceived Spirituality to post-traumatic growth.

First, it seems to be confirmed that, although related, they are not part of a single concept, and that lay people can distinguish perfectly well between the two and relate to the sacred in different ways, scoring higher in Perceived Spirituality than in Perceived Religiosity. Participants in our sample did not report being religious without being spiritual, and 20% said they were neither religious nor spiritual. The percentage of R-S- is lower in our study compared to other European countries such as Germany (Büssing et al., 2007 (for further analysis of the cultural differences in the meanings of these groupings consult Keller et al., 2013). The majority (31.7%) defined themselves as both religious and spiritual. Our subjects do not appear to see religiosity and spirituality as opposing or incompatible; spirituality is understood as an essential function of religion. R + S + subjects seem to have trust in a religious source as part of their spiritual quest, with this group being the one which had higher scores in growth and meaning. These results coincide with several studies which find psychological functioning is better in the R+ S+ group and worse in R− S+ (Schnell, 2012; Vittengl, 2018), which has been interpreted as “vulnerable people who are seeking existential meaning for their lives” (King et al., 2013, p. 161).

Second, the data show that each plays a different role in post-traumatic growth. Of particular interest is the fact that Perceived Spirituality broadly coincides with meaning in predicting growth, especially social and interpersonal, suggesting that it is through meaning that Perceived Spirituality influences these types of growth after traumatic situations. These data are compatible with those found by Büssing et al. (2007) in patients with chronic diseases: those with S+ reported greater search for meaningful support, as our study also appears to show. However, in the case of personal growth, it seems that Perceived Spirituality has a different function from that of Perceived Religiosity, and one that goes beyond meaning since it is only Perceived Spirituality that predicts such growth. Perhaps the resource of being able to take a positive view of situations, associated with S+ (Büssing et al., 2007), partly explains this result.

Perceived Religiosity, on the other hand, seems to contribute other significant values and models in addition to meaning, which facilitate social and interpersonal growth in the face of traumatic and life-threatening situations. According to Pargament (1997), religious coping contributes something special that is particularly interesting when responding to situations in which the subject comes face to face with their limits, and where their strength and control are confronted with their vulnerability and finitude, such as the situation caused by the coronavirus. Several authors have highlighted the social support function of religious participation (Shaw et al., 2005; López et al., 2015); indeed, López et al. showed that the greater the support of the religious community perceived by the participants, the greater the degree of post-traumatic growth. It would be interesting to explore the different functions that both variables, Perceived Religiosity and Perceived Spirituality, fulfill in traumatic situations.

This study has some limitations. First, social desirability was not controlled for, which could have affected the scores obtained for variables. Furthermore, although the sample is very large, it is not a representative sample. Although the distribution in age ranges is generally similar to that of the population of Madrid, some differences may have influenced results: our subjects perceived they are better off economically, and a higher percentage had university degrees than the general population. Perhaps, this results in a greater capacity for reflection and reasoning which, in turn, facilitates the experience of transcendence and meaning. Finally, our measure of religiosity and spirituality was global and self-rated, but religiosity has different meanings for different participants. This study did not explore its different dimensions (e.g., participation in religious practices and intrinsic religiosity), which makes it difficult to interpret our results. Future qualitative studies are necessary to reveal how lay people understand these constructs. Finally, we did not include other possible variables which could also influence growth, so our conclusions should be treated with caution. For example, with regard to social isolation, we did not take into account that people living alone have experienced a situation of greater social isolation.

However, our results clearly reflect the interest in maintaining the distinction between the two concepts, their different roles in traumatic growth and the different dimensions that each has an effect on. Finally, it confirms the importance of meaning in post-traumatic growth, especially the dimension of life goals and purposes. Even in situations as difficult as the one experienced, with the immediate threat of death and disease, during a strict lockdown, surrounded by pain and fear, it is possible, and more necessary than ever, that people reflect on purposes and goals in life, the experience of transcendence and meaning, and social support. Ultimately, our study reminds us that human beings are greater and stronger than their fear and pain.

## Data Availability Statement

The raw data supporting the conclusions of this article will be made available by the authors, without undue reservation.

## Ethics Statement

Ethical review and approval was not required for the study on human participants in accordance with the local legislation and institutional requirements. The patients/participants provided their written informed consent to participate in this study.

## Author Contributions

MP-U developed the study concept and study design. RJ substantially contributed to the study concept and performed the data analysis and interpretation. MP-U drafted the manuscript. Both authors approved the final version of the manuscript and also agreed to be accountable for all aspects of the work.

## Conflict of Interest

The authors declare that the research was conducted in the absence of any commercial or financial relationships that could be construed as a potential conflict of interest.

## Footnotes

1 https://coronavirus.jhu.edu/map.html

2 http://www.interior.gob.es/prensa/noticias/-/asset_publisher/GHU8Ap6ztgsg/content/id/11918219

3 https://proyectogaudete.com/covid19/
