# Supplementary material for: Finding Meaning in Hell. The Role of Meaning, Religiosity and Spirituality in Posttraumatic Growth During the Coronavirus Crisis in Spain
Source: Front Psychol. 2020 Nov 5;11:567836. doi: 10.3389/fpsyg.2020.567836 (PMC7674589; doi:10.3389/fpsyg.2020.567836)
Supplement: Supplementary file 1 [file Table_1.docx]

| **Supplementary Table 1.** Lineal Regression of Personal Growth on meaning and religiosity/spirituality *(controlled by age, sex and impact of COVID-19)*. | | | | | | |
| --- | --- | --- | --- | --- | --- | --- |
|  |  | Personal growth | | | 95 % CI | |
| Predictor | ΔR^2^ | β | se | Lower limit | | Upper  limit |
| Step 1 | .030** |  |  |  | |  |
| Age |  | .03 | .11 | -.02 | | .26 |
| Sex |  | 2.15** | .37 | 1.46 | | 2.94 |
| Step 2 | .012* |  |  |  | |  |
| Diagnosed |  | .557* | .24 | .10 | | 1.07 |
| Sibling hospital |  | -.18 | .57 | -1.26 | | .94 |
| Sibling IUC |  | -.17 | .75 | -1.65 | | 1.32 |
| Familiar death |  | .93* | .39 | .16 | | 1.73 |
| Sibling death |  | .28 | .63 | -1.05 | | 1.46 |
| Step 3 | .038*** |  |  |  | |  |
| SSV |  | .01 | .04 | -.08 | | .09 |
| MPV |  | .28*** | .06 | .16 | | .41 |
| Step 4 | .020*** |  |  |  | |  |
| Spirituality |  | .45* | .22 | .02 | | .88 |
| Religiosity |  | .34 | .17 | -.01 | | .67 |
| Total R^2^ | .101*** |  |  |  | |  |
