# Supplementary material for: Finding Meaning in Hell. The Role of Meaning, Religiosity and Spirituality in Posttraumatic Growth During the Coronavirus Crisis in Spain
Source: Front Psychol. 2020 Nov 5;11:567836. doi: 10.3389/fpsyg.2020.567836 (PMC7674589; doi:10.3389/fpsyg.2020.567836)
Supplement: Supplementary file 2 [file Table_2.docx]

| **Supplementary Table 2.** Lineal Regression of Interpersonal Growth on meaning and religiosity/spirituality *(controlled by age, sex and impact of COVID-19)*. | | | | | | |
| --- | --- | --- | --- | --- | --- | --- |
|  |  | Interpersonal growth | | | 95 % CI | |
| Predictor | ΔR^2^ | β | se | Lower limit | | Upper  limit |
| Step 1 | .010** |  |  |  | |  |
| Age |  | .18 | .10 | -.03 | | .37 |
| Sex |  | 1.18** | .37 | .43 | | 1.87 |
| Step 2 | .017** |  |  |  | |  |
| Diagnosed |  | .60* | .25 | .10 | | 1.08 |
| Sibling hospital |  | .42 | .54 | -.68 | | 1.46 |
| Sibling IUC |  | -1.15 | .78 | -2.72 | | .30 |
| Familiar death |  | 1.11** | .37 | .38 | | 1.81 |
| Sibling death |  | -.24 | .65 | -1.04 | | 1.60 |
| Step 3.1 | .069*** |  |  |  | |  |
| SSV |  | -.14 | .05 | -.10 | | .07 |
| MPV |  | .40*** | .07 | .26 | | .53 |
| Step 4.1 | .017*** |  |  |  | |  |
| Spirituality |  | .31 | .22 | -.11 | | .74 |
| Religiosity |  | .37* | .16 | .04 | | .68 |
| Total R^2^ | .113*** |  |  |  | |  |
