# Supplementary material for: Finding Meaning in Hell. The Role of Meaning, Religiosity and Spirituality in Posttraumatic Growth During the Coronavirus Crisis in Spain
Source: Front Psychol. 2020 Nov 5;11:567836. doi: 10.3389/fpsyg.2020.567836 (PMC7674589; doi:10.3389/fpsyg.2020.567836)
Supplement: Supplementary file 3 [file Table_3.DOCX]

| **Supplementary Table 3.** Lineal Regression of Social Growth on meaning and religiosity/spirituality *(controlled by age, sex and impact of COVID-19)*. | | | | | | |
| --- | --- | --- | --- | --- | --- | --- |
|  |  | Social growth | | | 95 % CI | |
| Predictor | ΔR^2^ | β | se | Lower limit | | Upper  limit |
| Step 1 | .026*** |  |  |  | |  |
| Age |  | .58** | .12 | .32 | | .81 |
| Sex |  | 1.35** | .45 | .49 | | 2.22 |
| Step 2 | .025* |  |  |  | |  |
| Diagnosed |  | .61* | .60 | .12 | | 1.07 |
| Siblings hospital |  | .88 | .58 | -.30 | | 1.98 |
| Siblings IUC |  | -.91 | .78 | -2.39 | | .63 |
| Familiar death |  | 1.80*** | .41 | 1.02 | | 2.67 |
| Sibling death |  | -.45 | .66 | -1.70 | | .81 |
| Step 3 | .074*** |  |  |  | |  |
| SSV |  | -.02 | .05 | -.11 | | .09 |
| MPV |  | .47*** | .07 | .32 | | .63 |
| Step 4 | .024*** |  |  |  | |  |
| Spirituality |  | .16 | .25 | -.33 | | .69 |
| Religiosity |  | .67*** | .19 | .29 | | 1.03 |
| Total R^2^ | .150*** |  |  |  | |  |
