# Supplementary material for: Finding Meaning in Hell. The Role of Meaning, Religiosity and Spirituality in Posttraumatic Growth During the Coronavirus Crisis in Spain
Source: Front Psychol. 2020 Nov 5;11:567836. doi: 10.3389/fpsyg.2020.567836 (PMC7674589; doi:10.3389/fpsyg.2020.567836)
Supplement: Supplementary file 4 [file Table_4.DOCX]

| **Supplementary Table 4.** Pearson Correlations. Meaning, Growth, Spirituality and Religiosity | | | | | | | | | |
| --- | --- | --- | --- | --- | --- | --- | --- | --- | --- |
|  | | 1 | 2 | 3 | 4 | 5 | 6 | 7 | 8 |
| 1. Total meaning |  | 1 |  |  |  |  |  |  |  |
| 1. SSL |  | .962^**^ |  |  |  |  |  |  |  |
| 1. GPL |  | .892^**^ | .735^**^ |  |  |  |  |  |  |
| 1. Total growth |  | .266^**^ | .220^**^ | .295^**^ |  |  |  |  |  |
| 1. Personal growth |  | .190^**^ | .157^**^ | .216^**^ | .874^**^ |  |  |  |  |
| 1. Interpersonal growth |  | .247^**^ | .202^**^ | .274^**^ | .916^**^ | .725^**^ |  |  |  |
| 1. Social growth |  | .270^**^ | .224^**^ | .293^**^ | .858^**^ | .579^**^ | .701^**^ |  |  |
| 1. Spirituality |  | .305^**^ | .277^**^ | .296^**^ | .226^**^ | .205^**^ | .193^**^ | .200^**^ |  |
| 1. Religiosity |  | .259^**^ | .242^**^ | .244^**^ | .224^**^ | .178^**^ | .186^**^ | .230^**^ | .629^**^ |
